# Vaccine Virus

**Published:** 1880-12

**Authors:** 


Vaccine Virus.—Dr. Stevens, of London, and Dr. Atlee, who have both had an immense experience in vaccination, declare there has not been the slightest deterioration in the efficacy of the humanized lymph.—*St. Louis Courier of Medicine.*

